# 경피적 관상동맥중재술을 받은 국내 환자의 건강관리 자기효능감과 회복탄력성이 치료지시이행에 미치는 영향요인: 횡단적 조사 연구

**DOI:** 10.7586/jkbns.25.013

**Published:** 2025-05-20

**Authors:** Mi-Ra Jung, Eun Jeong, Kyung Sim Lee, Jang Hyun Cho

**Affiliations:** 1 Department of Nursing, Hanyeong University, Yeosu, Korea; 1 한영대학교 간호학과; 2 Department of Nursing, Suncheon Jeil University, Suncheon, Korea; 2 순천제일대학교 간호학과; 3 Division of Cardiology, Department of Internal Medicine, St. Carollo Hospital, Suncheon, Korea; 3 성가롤로병원 순환기내과

**Keywords:** Self efficacy, Resilience, psychological, Population health management, Patient compliance, Percutaneous coronary intervention, 자기효능감, 회복탄력성, 건강관리, 환자 지시이행, 관상동맥 중재

## Abstract

**Purpose:**

This study aimed to identify factors influencing treatment compliance in Korean patients undergoing percutaneous coronary intervention.

**Methods:**

The study included 130 patients who underwent percutaneous coronary intervention at St. Carollo Hospital. Data were collected using self-administered questionnaires from March 21, 2022 to May 30, 2023, and analyzed using descriptive statistics, the t-test, analysis of variance, the Scheffé test, Pearson correlation coefficients, and multiple regression in SPSS version 26.0.

**Results:**

Resilience (β = .59, *p* < .001), alcohol drinking (β = −.18, *p* = .005), and the diagnosis (β = .13, *p* = .040) accounted for 45.0% of the variance in treatment compliance in patients undergoing percutaneous coronary intervention.

**Conclusion:**

These results indicate that it is necessary to explore strategies for developing and implementing programs that can effectively enhance resilience in patients who have undergone percutaneous coronary intervention.

## 서론

### 1. 연구의 필요성

통계청에 따르면, 우리나라 사망원인 2위인 심장질환은 인구 10만명 당 2013년 50.2명에서 2023년 64.8명으로 29.2% 증가 추세를 보이고 있다[1]. 심혈관 질환 중 관상동맥질환(Coronary Artery Disease)은 관상동맥 내에 죽상경화성 병변이 진행되어 혈관이 좁아지거나 폐쇄되어 발생하는 질환으로 협심증과 심근경색증을 유발시킨다[2].

관상동맥질환의 일반적인 치료방법으로는 위험요인과 증상을 조절하는 약물요법, 허혈과 경색의 진행을 예방하기 위한 경피적 관상동맥중재술(Percutaneous Coronary Intervention) 및 관상동맥우회술 등이 있으며[3], 그 중 관상동맥중재술은 관상동맥의 협착 부위를 치료하기 위하여 전 세계적으로 광범위하게 시행되고 있는 표준치료법으로 국내에서도 널리 시행되고 있다[4]. 경피적 관상동맥중재술은 스텐트 삽입과 풍선 확장을 포함하는 것으로, 관상동맥의 협착으로 인한 허혈성 심근에 혈류를 증가시키며, 수술에 비해 초기 의료비용 절감, 빠른 업무 복귀 및 활동적인 일상을 재개할 수 있는 이점이 있다[5]. 경피적 관상동맥중재술은 질환의 이환율 증가와 조기 발견, 중재술의 발전으로 매년 시행 건수가 늘어, 국내의 경우 스텐트삽입술을 제외한 경피적 관상동맥중재술은 2019년 8,710건에서 2021년 9,658건, 2023년 16,841건으로 연평균 증감률은 17.9%를 보였다[6]. 경피적 관상동맥중재술 시행 후에도 경과에 대한 관심과 적극적 관리가 필요하며, 운동과 식이요법, 올바른 약물복용 등으로 재협착 예방 및 기능 회복이 중요하며 적절한 치료지시이행은 심혈관 사건 발생이 감소하고 평균 기대 수명이 연장되는 것으로 나타났다[7].

치료지시이행은 환자가 임상적 처방에 잘 순응하는 것으로 증상이 있을 때 진찰을 받는 것, 치료를 꾸준히 계속하는 것, 위험을 줄이기 위해 생활습관을 변경하는 것으로[8] 하위영역의 식이, 운동, 약물요법, 기호식품의 제한, 병원방문, 체중관리, 신체적, 정신적 안정을 포함하고 있다. 특히 경피적 관상동맥중재술을 받은 환자는 완치가 아닌 증상 완화를 목표로 치료를 받으며, 시술 후 즉각적인 임상적 호전으로 재원 기간이 짧고 일상 복귀가 빠른 만큼, 퇴원 후에도 관상동맥질환자들의 경우 스스로 치료지시이행을 잘하도록 돕는 것이 중요하다[9]. 따라서 관상동맥질환자의 생활습관 및 건강행태 등의 질병관리에 필요한 건강행위를 잘 이행함으로써 삶의 질을 높이며[10] 경피적 관상동맥중재술을 받은 환자의 건강행태 및 약물요법 등의 건강행위에 잘 순응하여 치료지시이행을 높이는 전략이 필요하다. 관상동맥질환자의 치료지시이행을 위해서는 동기부여와 건강행위를 위한 행동변화에 가장 직접적인 영향을 주는 요인으로는 자기효능감이었다[11]. 자기효능감은 관상동맥질환자의 생활양식 변화 및 자기관리 이행을 증진시키기 위한 전략으로 나타나 건강행위 이행을 촉진하는데 필요한 요인으로 작용하였다[12].

건강관리 자기효능감은 스스로 건강관리를 실행할 수 있다는 자신감으로 건강관리의 필요성을 느낀 후 생각한 것을 실제 행동으로 실천할 수 있는 믿음을 의미한다[13]. 건강관리 자기효능감의 하위영역으로 운동관리, 질병관리, 정서관리, 영양관리, 스트레스관리, 건강관리행동으로 구분된다[14]. 특히 개인이 자신의 건강을 관리할 수 있는 건강실천행위에 자기효능감이 높을수록 질병관리에 대한 잠재능력이 높아지는 건강관리 자기효능감을 갖는 것이 필요하다[15]. 일반적 수준의 자기효능감이 아닌 건강관리에 대한 자신감을 나타내는 건강지식을 아는 것을 넘어 실제 행동으로 움직이기 위해 높은 건강관리 자기효능감[14]은 경피적 관상동맥중재술을 받은 환자에게 건강행위와 건강관리에 대한 자신감을 향상시킴으로써 치료지시이행을 높이는 것을 확인할 필요가 있다. 이러한 관상동맥질환자의 심리적 요인들은 만성질환의 치료에 영향을 주므로 신체적, 심리적 변화를 극복하고 지속적인 자가간호 행위를 증진 시킬 수 있는 회복탄력성을 확인하는 것은 치료지시이행을 높이기 위한 수단으로 중요하다.

회복탄력성(Resilience)는 극심한 위험 상황이나 만성적 스트레스에 따르는 외상에도 불구하고 성공적 적응을 이룰 수 있는 능력을 의미한다[16]. 즉 개인이 직면한 역경에 적응하고 오히려 성장을 가능하게 하는 개인의 사회 심리적 특성 및 질병, 위기, 불행 등으로부터 빨리 회복하여 이겨내는 힘을 말한다[17]. 특히 심혈관질환자의 회복력은 개인적 차원에서 긍정적 회복신념과 재건력, 조절능력을 가지고 관계적 차원에서 지지적 자원과의 긍정적 상호작용을 통해 심장병으로부터 회복하는 힘이라 할 수 있다[18]. 선행연구에 따르면, 관상동맥질환자의 회복탄력성은 심혈관질환의 회복과정에서 중요한 요인으로 작용하였으며, 회복탄력성이 높은 환자는 자기효능감과 건강행위를 높이는 것으로 나타났다[19]. 또한 외래를 방문하는 관상동맥중재술을 받은 노인을 대상으로 건강행위 이행에 회복탄력성이 가장 큰 영향을 미치는 것으로 확인되었다[20]. 아울러 위기에 반응하여 이를 감당하고 스스로를 조절하며 성장하는 힘으로써, 처음 관상동맥질환을 진단받은 대상자에게 회복탄력성을 높이는 것은 치료지시이행을 높이기 위한 중요한 변인이었다[12,21] 이에 경피적 관상동맥중재술을 받은 환자의 회복탄력성을 살펴보고 치료지시이행에 미치는 영향을 확인할 필요가 있다. 기존 선행연구에서, 관상동맥질환자를 대상으로 한 치료지시이행에 관상동맥질환 위험정도와 혈관탄성[9], 자기효능감, 낙관성 및 회복탄력성[12], D유형 성격과 극복력[21] 등으로 나타났지만, 경피적 관상동맥중재술을 받은 환자의 치료지시이행에 미치는 영향을 좀 더 확인할 필요가 있다.

지금까지 경피적 관상동맥중재술을 받은 환자의 선행연구를 살펴보면, 관상동맥질환 위험정도와 혈관탄성의 관계에서 치료지시이행의 매개효과[9], 자기효능감과 회복력[19], 건강행위이행[10,22], 건강정보 이해능력, 자기효능감 및 사회적 지지가 건강관련 삶의 질[23] 등으로 연구되어왔으며, 경피적 관상동맥중재술을 받은 환자의 건강관리에 대한 자신감으로 건강관리 자기효능감과 회복탄력성이 치료지시이행과의 관계를 함께 고려한 연구는 없었다. 이에 본 연구에서는 경피적 관상동맥중재술을 받은 환자의 건강관리 자기효능감, 회복탄력성이 치료지시이행에 미치는 영향을 파악함으로써 경피적 관상동맥중재술을 받은 환자의 치료지시이행을 증진 시키는데 효과적인 간호 중재 방안을 마련하고자 시도되었다.

### 2. 연구 목적

본 연구의 목적은 경피적 관상동맥중재술을 받은 환자의 치료지시이행에 미치는 영향을 규명하기 위함이며, 구체적인 목적은 다음과 같다.

첫째, 대상자의 일반적 특성과 일반적 특성에 따른 치료지시이행의 차이를 파악한다.

둘째, 대상자의 건강관리 자기효능감, 회복탄력성과 치료지시이행의 정도를 파악한다.

셋째, 대상자의 건강관리 자기효능감, 회복탄력성, 치료지시이행의 상관관계를 파악한다.

넷째, 대상자의 치료지시이행에 영향을 미치는 요인을 규명한다.

## 연구 방법

### 1. 연구 설계

본 연구는 관상동맥중재술을 받은 환자의 치료지시이행 정도와 이에 영향을 미치는 요인을 규명하기 위한 서술적 조사연구이다.

### 2. 연구 대상

본 연구는 전라남도에 소재한 성가롤로병원 순환기내과에서 관상동맥질환을 진단받고 경피적 관상동맥중재술을 시행한 후 외래를 통해 추후 관리를 받고 있는 대상자를 편의 추출하였다. 구체적인 대상자 선정기준은 첫째, 관상동맥질환을 진단받고 외래를 통해 추후 관리 중인 대상자, 둘째, 경피적 관상동맥중재술 시행자, 셋째, 본 연구에 자발적으로 참여하고자 동의한 자, 넷째, 설문지를 읽고 응답할 수 있거나 의사소통이 가능한 자이며, 기질적 뇌질환이나 정신질환이 있어 의식이 불명확한 자는 대상자 선정기준에서 제외하였다.

표본 수는 G*Power program 3.1.9.7[24] 프로그램을 이용하여 관상동맥질환을 진단받고 경피적 관상동맥중재술을 시행한 자를 대상으로 한 선행연구[10]를 참고하여 효과크기를 산출하였다. 다중회귀분석에 필요한 적정 표본수를 산출한 결과, 유의수준 .05, 효과크기 .15, 검정력 .80, 예측 변인 12개를 기준으로 대상자 수를 127명으로 도출하였다. 탈락률 10%를 고려하여 총 142부의 설문지를 배부하였으며, 이 중 응답이 불충분한 설문지를 제외한 130부를 최종 분석에 활용하였다.

### 3. 연구 도구

#### 1) 일반적 특성

대상자의 성별, 연령, 최종학력, 직업 유무, 월수입, 동거형태, 흡연 여부, 음주 유무, 운동, 키, 몸무게, 진단명, 최초 진단 시기, 경피적 관상동맥중재술 시기, 동반질환, 건강상태를 확인하는 총 16문항으로 구성하였다. 체질량지수(body mass index)는 체중(kg)을 키의 제곱(m^2^)으로 나눈 값을 의미하며, 저체중 18.4 kg/m^2^ 이하, 정상체중 18.5~22.9 kg/m^2^, 과체중 23~24.9 kg/m^2^, 비만 25 kg/m^2^ 이상으로 분류한다[25].

#### 2) 건강관리 자기효능감

건강관리 자기효능감은 건강관리에 도움이 되며 건강을 유지하여 행위를 파악하고 실천할 수 있는 믿음의 정도를 의미하며[14], Becker 등[13]이 개발한 후, 한국어판으로 타당도를 검증한 Lee 등[14]의 총 24문항 도구를 사용하였다. 이 도구는 운동관리 8문항, 질병관리 4문항, 정서관리 3문항, 영양관리 3문항, 스트레스관리 3문항, 건강관리행동 3문항의 6개 하위영역으로 구성되었으며, Likert 5점 척도로 점수가 높을수록 건강관리 자기효능감이 높음을 의미한다. 선행연구 Lee 등[14]의 신뢰도 Cronbach’s ⍺는 .86였으며, 본 연구에서는 Cronbach’s ⍺는 . 89였다.

#### 3) 회복탄력성

회복탄력성은 극심한 위험 상황이나 만성적 스트레스에 따르는 외상에도 불구하고 성공적 적응을 이룰 수 있는 능력을 의미하며[16], 본 연구에서는 Shin[26]이 심혈관질환 회복력 측정도구를 개발하기 위해 급성관상동맥증후군 대상자로 신뢰도와 타당도를 검증한 총 25문항의 도구를 사용하였다. 이 도구는 지지적 관계 6문항, 건강계획 실천능력 4문항, 조절적 5문항, 긍정적 태도 4문항, 회복신념 2문항, 의료진과의 관계 2문항, 극복 자신감 2문항으로 구성되었으며, Likert 5점 척도로 점수가 높을수록 회복탄력성이 높음을 의미한다. Shin[26]의 신뢰도 Cronbach’s ⍺는 .84였으며, 본 연구에서는 Cronbach’s ⍺는 .92였다.

#### 4) 치료지시이행

치료지시이행은 환자가 의료인 및 의료기관이 지시하는 임상적 처방에 잘 순응하는 것으로 증상이 있을 때 진찰을 받는 것, 치료를 꾸준히 계속하는 것, 위험을 줄이기 위해 생활습관을 변경하는 것을 의미하며[8], 관상동맥중재술을 받고 재협착을 막기 위해 지켜야 할 Shin[27]의 도구 총 16문항을 사용하였다. 이 도구는 식이 3문항, 운동 2문항, 약물요법 2문항, 기호식품의 제한 2문항, 병원방문 1문항, 체중관리 1문항, 신체적, 정신적 안정 5문항으로 구성되었으며, Likert 4점 척도로 점수가 높을수록 치료지시이행이 높음을 의미한다. Shin[27]의 신뢰도 Cronbach’s ⍺는 .75였으며, 본 연구에서는 Cronbach’s ⍺는 .75였다.

### 4. 자료 수집

자료수집 기간은 2022년 3월 21일부터 2023년 5월 30일까지였으며, 전라남도에 소재한 성가롤로병원 순환기내과에서 관상동맥질환을 진단받고 경피적 관상동맥중재술을 시행한 후 외래를 통해 추후관리를 받고 있는 대상자에게 외래 진료가 끝난 후 연구자가 직접 설문조사를 시행하였다. 연구참여를 자발적으로 허락한 대상자에게 서면동의를 받고 설문조사를 시행하였으며 응답내용은 익명으로 처리하며 응답결과는 오직 연구목적으로만 사용할 것을 설명하였고 연구진행 시 언제든지 참가동의를 철회할 수 있음을 설명하였다. 수집된 설문지는 밀봉된 봉투에 넣어 회수하였으며 설문지에 소요되는 시간은 15-20분 정도였다.

### 5. 자료 분석

SPSS version 26.0 (IBM Corp., Armonk, NY, USA) 프로그램을 이용하였으며, 통계적 유의성은 *p* < .05로 하였다. 구체적인 자료분석방법은 다음과 같다.

대상자의 일반적 특성, 건강관리 자기효능감, 회복탄력성과 치료지시이행 정도는 기술통계를 이용하여 분석하였으며, 일반적 특성에 따른 치료지시이행의 차이는 t-test, one-way ANOVA, 사후검증은 Scheffé test로 하였다. 대상자의 건강관리 자기효능감, 회복탄력성과 치료지시이행의 상관관계는 Pearson correlation coefficient로 분석하였으며, 대상자의 치료지시이행에 영향력이 있는 변수를 파악하고자 단계적 다중회귀분석(stepwise multiple regression)을 시행하였다.

### 6. 윤리적 고려

본 연구는 성가롤로병원의 기관생명윤리심의위원회 승인(IRB No. SCH2022-0153)을 받은 후 진행하였다. 본 연구에 참여하기로 동의한 대상자에게 연구목적과 내용을 충분하게 설명한 후 서면동의를 받고 참여한 대상자에게 기념품을 제공하였다.

## 연구 결과

### 1. 일반적 특성

연구대상자의 성별은 남성 115명(88.5%), 여성 15명(11.5%)으로 나타났다. 평균 연령은 63.55 ± 7.87세였으며, ‘60~69세’ 73명(56.2%), ‘70세 이상’ 29명(22.3%), ‘50~59세’ 19명(14.6%), ‘50세 미만’ 9명(6.9%)순으로 나타났다. 직업이 없는 경우가 87명(66.9%)으로 직업이 있는 경우 43명(33.1%)보다 많은 것으로 나타났으며, 동거가족에서 ‘배우자와 사는 경우’가 73명(56.2%), ‘가족과 사는 경우’가 42명(32.3%), ‘혼자 사는 경우’는 15명(11.5%) 순으로 나타났다. 흡연유무에서 ‘담배를 피우지 않음’이 58명(44.6%)으로 ‘현재 금연’ 47명(36.2%)과 ‘흡연’ 25명(19.2%)보다 많은 것으로 나타났고, 술을 마시지 않은 경우가 84명(64.6%)으로 마시는 경우 46명(35.4%)보다 많은 것으로 나타났다. 운동을 불규칙적으로 하는 경우가 70명(53.8%)으로 규칙적으로 하는 경우 53명(40.8%)보다 많은 것으로 나타났으며, 체질량지수는 ‘비만’인 경우 58명(44.6%), ‘과체중’이 45명(34.6%), ‘정상체중’이 27명(20.8%) 순으로 나타났다. 경피적 관상동맥중재술 시기는 ‘1~5년’ 78명(60.0%), ‘6~10년’ 40명(30.8%)으로 많았으며, 주관적 건강상태에서 ‘보통’ 71명(54.6%), ‘좋음’ 41명(31.5%), ‘나쁨’ 18명(13.9%) 순으로 나타났다. 진단명은 ‘협심증’ 86명(66.2%), ‘심근경색’ 44명(33.8%)이었으며, 질병기간은 ‘2~5년’ 53명(40.8%), ‘6~10년’ 39명(30.0%), ‘1년 이내’ 24명(18.4%), ‘11년 이상’은 14명(10.8%)이었다. 동반질환은 ‘고혈압’ 58명(44.6%), ‘당뇨’ 30명(23.1%), ‘고지혈증’ 16명(12.3%)으로 가장 많았다(Table 1).

대상자의 치료지시이행은 일반적 특성 중 흡연유무(F = 11.93, *p* < .001), 음주유무(t = 3.88, *p* < .001), 운동(F = 9.28, *p* < .001), 경피적 관상동맥중재술 시기(F = 3.51, *p* = .033), 주관적 건강상태(F = 7.51, *p* < .001), 진단명(t = 2.08, *p* = .041)에 따라 유의한 차이가 있는 것으로 나타났다. 사후검증결과 흡연유무에서 ‘담배를 피우지 않음’과 ‘현재 금연’이 ‘흡연’보다, 운동에서 ‘규칙적인 운동’이 ‘안함’과 ‘불규칙인 운동’보다, 경피적 관상동맥중재술 시기에서 ‘6~10년’이 ‘1~5년’보다, 주관적 건강상태에서 ‘좋음’이 ‘나쁨’과 ‘보통’보다 높은 것으로 나타났다(Table 1).

### 2. 건강관리 자기효능감, 회복탄력성과 치료지시이행 정도

대상자의 건강관리 자기효능감은 평균 5점 만점에 3.75 ± 0.49점, 회복탄력성은 3.66 ± 0.45점, 치료지시이행 정도는 평균 4점 만점에 3.00 ± 0.35점으로 나타났다(Table 2).

### 3. 대상자의 건강관리 자기효능감, 회복탄력성과 치료지시이행 간 상관관계

치료지시이행은 건강관리 자기효능감(r = .46, *p* < .001), 회복탄력성(r = .63, *p* < .001)과 유의한 양의 상관관계를 보였다(Table 3).

### 4. 대상자의 치료지시이행에 영향을 미치는 요인

대상자의 치료지시이행에 영향을 미치는 요인을 알아보기 위해 단계적 다중회귀분석을 시행하였다.투입된 변수는 치료지시이행과 통계적으로 유의한 차이를 보이거나 유의한 상관관계를 나타낸 일반적 특성(흡연 여부, 음주유무, 운동, 경피적 관상동맥중재술 시기, 주관적 건강상태, 진단명)과 건강관리 자기효능감, 회복탄력성이었다. 흡연 여부, 음주유무, 운동, 경피적 관상동맥중재술 시기, 주관적 건강상태, 진단명은 더미변수로 처리하여 투입하였다.

단계적 다중회귀분석 결과, 회귀모형은 통계적으로 유의하게 나타났다(F = 4.30, *p* = .040). Durbin-Watson 통계량은 2.10의 값을 보여 잔차의 독립성 가정에 문제가 없는 것으로 확인되었고, 정규성도 확인하였다. 다중공선성을 확인한 결과 공차한계(tolerance)는 .96∼.99, 분산팽창인자(variance inflation factor)는 1.01-1.04로 10을 넘지 않아 다중공선성의 문제는 없는 것으로 나타났다. 치료지시이행에 영향을 미치는 요인으로 회복탄력성이 높을수록 치료지시이행을 높게 하였고(β= .59, *p* < .001), 술을 먹는 사람은 안먹는 사람보다 치료지시이행을 낮게 하였고(β = −.18, *p* = .005), 진단명이 심근경색증인 사람이 협심증인 사람보다 치료지시이행을 높게 하였다(β = .13, *p* = .040). 상대적 영향력 정도는 회복탄력성이 가장 큰 것으로 나타났으며, 회복탄력성, 음주유무, 진단명을 모두 포함시켰을 때의 영향요인은 치료지시이행을 총 45.0%를 설명하는 것으로 나타났다(Table 4).

## 논의

본 연구는 경피적 관상동맥중재술을 받은 대상자의 치료지시이행에 영향을 미치는 요인을 규명함으로써 관상동맥질환자의 치료지시이행을 증진시킬 수 있는 요인을 통해 관상동맥질환자의 자가관리를 지속적으로 실천할 수 있는 방안을 모색하고자 시도되었다. 그 결과 경피적 관상동맥중재술을 받은 환자의 회복탄력성, 음주유무와 진단명이 치료지시이행에 영향을 미치는 것으로 확인되었다.

본 연구대상자의 치료지시이행 정도는 4점 만점에 평균 3.00 ± 0.35점으로 이는 동일한 도구를 사용한 Lee와 Sung[22]의 연구에서 3.11점으로 비슷하였으며, 이러한 결과는 경피적 관상동맥중재술 후 임상적 처방 지시에 잘 순응하여 장기적인 자기관리 도모의 치료지시이행은 중요한 요인으로 하위영역의 약물요법과 생활습관 개선지도 등의 이행을 높이기 위해 외래 방문하는 대상자에게 의료진은 주기적인 교육과 증상 모니터링 및 심리적 지지가 필요하다. 또한 대상자와 도구를 달리한 선행연구에서 고혈압환자의 치료지시이행은 3.19점으로 나타났으며[28], 본 연구결과 경피적 관상동맥중재술을 시행한 후 외래를 통해 정기적인 관리를 위해 추후 관리를 받고 있는 환자의 치료지시이행은 질병을 관리하고 치료하기 위해 지속적으로 수행되어야 하는 필수요인으로 치료지시이행을 향상시키기 위해 대상자와 목표설정 및 구체적인 계획을 세워 실행하도록 지지가 필요하다.

본 연구대상자의 건강관리 자기효능감 정도는 평균 3.75점(범위:1∼5점)으로 나타났다. 치료지시이행에 건강관리 자기효능감을 확인한 연구가 없어 직접 비교는 어렵지만 자기효능감이 높은 경우 특정행동을 실천하기 위한 노력과 지속성이 증가하게 되어 쉽게 포기하지 않게 되는 건강관리 자기효능감[14]이 필요하다. Kim과 Jung[11]의 연구에서 경피적 관상동맥중재술을 받은 환자에서 심장재활 프로그램 참여가 자기효능감을 높이는 것으로 Lee와 Sung[22]의 연구에서도 경피적 관상동맥중재술 환자의 자기효능감은 대표적인 심리적 요인으로 환자 스스로 회복에 대한 자신감의 신념으로 본 연구결과를 뒷받침하고 있다. 또한 대상자를 달리한 중소병원 간호사를 대상으로 한 선행연구[15]에서 건강관리 자기효능감은 3.84점으로 비슷한 결과를 보였으며, 일반적인 스트레스 관리 및 목표행동을 실행할 수 있다는 믿음의 건강관리에 자신감을 높이는 건강관리 자기효능감은 경피적 관상동맥중재술을 받은 환자에게 요구되는 변인으로 반복연구를 통해 알아볼 필요가 있겠다. 따라서 경피적 관상동맥중재술을 받은 후 높은 자기효능감은 스스로 건강에 대한 긍정적인 태도를 확립하고 질병의 치료에 대한 자신감을 향상시킴으로써 건강관리와 질병을 회복하는 과정에서 중요하게 파악해야 할 요소로 여겨진다.

본 연구대상자의 회복탄력성 정도는 평균 3.66 ± 0.45점(범위:1∼5점)으로 나타났다. 본 연구에서 경피적 관상동맥중재술을 시행한 지 1~5년이 지난 대상자가 60.0%인 반면에 Jeon과 Chang[20]의 연구에서 경피적 관상동맥중재술을 받은 12개월 이내 지역사회 거주 노인의 회복탄력성 정도는 3.88점으로 본 연구보다 높은 것으로 나타났으며, Jeong[12]의 연구에서 관상동맥조영술을 시행한 후 관상동맥질환을 진단받은 지 6개월 이내인 환자의 회복탄력성은 평균 3.63점으로 본 연구결과와 유사한 결과를 보여 경피적 관상동맥중재술의 시기에 따라 회복탄력성 정도가 차이가 있는 것으로 나타나 추후 경피적 관상동맥중재술 시기에 따른 연구가 필요할 것으로 보인다. 즉, 회복탄력성은 심혈관질환자의 회복과정에서 경험하는 신체적, 심리적 증상 및 건강증진행위에 중요한 변인으로 경피적 관상동맥중재술을 받은 시기를 고려하여 대상자에게 다양한 프로그램을 적용함으로 치료지시이행을 증진시키는 전략이 요구된다.

대상자의 일반적 특성에 따른 치료지시이행의 차이를 보면, 흡연유무, 음주유무, 운동, 경피적 관상동맥중재술 시기, 주관적 건강상태, 진단명에 따라 유의한 차이가 있는 것으로 나타났다. 이는 담배를 피우지 않고, 현재 금연, 규칙적인 운동이, 경피적 관상동맥중재술 후 6년 이상 경과 시 주관적 건강상태가 좋음이, 심근경색증보다 협심증이 치료지시이행이 높은 것으로 나타났다. 이는 선행연구에서 음주와 흡연을 하지 않는 경우가 치료지시이행이 높은 것[12,21]으로 같은 결과를 보였으며, 경피적 관상동맥중재술 후, 재발 및 합병증을 막기 위해 지속적이고 적극적인 건강관리는 생명과 직결되는 주요 사안으로 치료지시이행을 잘 지키는 것이 중요함을 시사한다. 경피적 관상동맥중재술 후 치료지시이행에 따른 연구는 활발히 이루어지지 않아 추후 연구를 통해 경피적 관상동맥중재술 시기에 따른 주관적 건강상태, 진단명, 연령대별, 입원과 외래 방문 환자 및 다양한 변인으로 치료지시이행을 확인할 필요가 있다.

본 연구대상자의 치료지시이행은 건강관리 자기효능감, 회복탄력성과 양의 상관관계가 있었다. 즉 건강관리 자기효능감과 회복탄력성이 높을수록 치료지시이행을 잘하는 것으로 건강관리 자기효능감을 비교한 선행연구가 없어 직접 비교가 어렵지만, Jeong[12]의 연구에서 자기효능감과 회복탄력성이 높을수록 치료지시이행을 높이는 것으로 본 연구결과와 비슷한 결과를 보였다. 이는 건강관리 자기효능감이 높은 대상자는 건강관리에 대한 자신감의 건강목표를 달성할 수 있다는 확신과 심혈관질환자의 건강실천능력과 회복신념에 대한 회복탄력성을 높이는 것이 경피적 관상동맥중재술을 받은 환자에게 지속적으로 치료지시이행을 더 잘 할 수 있는 요인이 될 수 있다.

본 연구결과, 경피적 관상동맥중재술을 받은 대상자의 치료지시이행에 유의한 영향을 미치는 요인으로 회복탄력성, 음주유무, 진단명으로 나타났다. 먼저 회복탄력성은 치료지시이행에 가장 큰 영향을 미치는 요인으로 확인되었다. 선행연구에 따르면, 관상동맥을 진단받은 1개월에서 6개월 사이의 대상자에게 회복력을 높이는 것은 치료지시이행을 높이게 하는 중요한 변인으로 회복탄력성은 치료지시이행에 미치는 영향요인이라고 한 선행연구[12]와 일치한다. 또한 관상동맥중재술을 받은 지역사회 거주 노인에서 건강행위이행에 회복탄력성이 유의미한 영향을 미치는 것으로 나타나 본 연구결과를 지지하였다[20]. 따라서 관상동맥질환은 경피적 관상동맥중재술 후에도 평생 건강관리가 필요한 만성질환으로, 이전의 적응 수준으로 회복하기 위한 회복탄력성이 신체적•정신적 건강과 질병 회복과정에서 중요한 개념이므로, 차별화된 교육 프로그램을 적용하여 치료지시 이행을 증진할 필요가 있다.

본 연구에서 음주유무는 치료지시이행에 미치는 두 번째 영향요인으로 술을 먹는 사람은 안먹는 사람보다 치료지시이행을 낮게 하는 것으로 나타났다. 이는 선행연구에서도 관상동맥질환자가 음주를 안 할수록 치료지시이행이 더 높은 것[12]으로 본 연구결과와 일치하였다. 따라서 음주유무가 치료지시이행에 영향을 미치는 요인으로 관상동맥질환자에게 교육을 통해 지속적 건강관리를 돕는 것이 중요하다. 마지막으로 진단유무에 따라 즉 심근경색증이 협심증 환자보다 치료지시이행이 높은 것으로 나타났으며 선행연구가 없어 직접비교는 어렵지만 질병의 중등도가 높을수록 치료지시이행에 더 많은 주의와 요구가 필요한 것으로 영향을 미친다고 볼 수 있다.

본 연구는 경피적 관상동맥중재술을 받은 환자의 치료지시이행에 영향을 미치는 요인을 확인하고 회복탄력성의 변수를 적용하여 경피적 관상동맥중재술 환자의 치료지시이행을 높이기 위한 프로그램 개발을 위한 기초자료를 마련하였다는데 그 의의가 있다. 자기효능감은 치료지시이행에 중요한 영향요인이었으나 반면, 건강관리에 대한 자신감을 높이는 건강관리 자기효능감은 치료지시이행에 지지 되지 않아 추후 후속연구가 요구된다.

이상의 결과를 종합해 볼 때, 경피적 관상동맥중재술을 받은 환자의 회복탄력성을 높이고 이를 통해 치료지시이행을 잘할 수 있도록 유도하기 위해서는 회복탄력성을 효과적으로 높일 수 있는 다양한 프로그램을 개발 및 적용시킬 방안을 모색할 필요가 있다. 또한 개별화된 맞춤형 치료계획 및 지속적인 환자의 모니터링과 건강한 생활습관 형성에 대한 집중관리와 교육이 경피적 관상동맥중재술 환자에게 치료지시이행을 높여 재발 및 합병증 예방에 도움이 될 것이라 기대된다.

## 결론

본 연구는 경피적 관상동맥중재술을 받은 환자로 외래를 통해 추후 관리 중인 관상동맥질환자의 치료지시이행에 영향을 미치는 요인을 파악하여 관상동맥질환자의 치료지시이행을 증진 시킬 수 있는 프로그램 개발을 위한 기초자료를 제공하고자 시도되었다. 연구결과 경피적 관상동맥중재술을 받은 환자의 치료지시이행 영향요인은 회복탄력성, 음주유무, 진단명 순으로 나타났다. 관상동맥질환은 장기적인 관리가 필요한 질환이므로 회복하여 이겨내는 힘의 회복탄력성 증진 프로그램 개발 통해 적극적인 참여를 유도하여야 하며, 생활습관 및 지속적 모니터링 유지•관리를 통해 치료지시이행을 강화하여야 할 것이다.

이상의 결과를 바탕으로 다음과 같이 제언하고자 한다. 본 연구는 일개 지역 종합병원의 경피적 관상동맥중재술을 받은 대상으로 하였으므로 다양한 인구 사회학적, 질병관련 특성을 고려한 후속연구 제언한다. 또한 본 연구결과를 바탕으로 경피적 관상동맥중재술을 받은 환자의 치료지시이행을 높을 수 있는 회복탄력성 및 다양한 치료지시이행 증진 프로그램의 개발과 그 효과를 검증하는 연구를 제언한다.

## Figures and Tables

**Table 1.** Differences in Treatment Compliance According to Characteristics of Participants and Demographic Factors (N = 130)

Characteristics	Categories	n (%)	M ± SD	t or F (*p*)
Sex	Men	115 (88.5)	2.99 ± 0.35	−1.57 (.118)
	Women	15 (11.5)	3.14 ± 0.29	
Age (yr)	< 50	9 (6.9)	2.72 ± 0.29	2.55 (.058)
	50~59	19 (14.6)	2.97 ± 0.38	
	60~69	73 (56.2)	3.02 ± 0.37	
	≥ 70	29 (22.3)	3.07 ± 0.20	
Education level	≤ High school	81 (62.3)	3.01 ± 0.33	0.20 (.837)
	≥ College	49 (37.7)	3.00 ± 0.38	
Occupation	No	87 (66.9)	2.97 ± 0.37	−1.54 (.124)
	Yes	43 (33.1)	3.07 ± 0.29	
Monthly income (10,000 won)	None	12 (9.2)	3.06 ± 0.31	0.63 (.638)
	200~300	35 (27.0)	3.02 ± 0.32	
	301~399	38 (29.2)	2.94 ± 0.36	
	400~500	19 (14.6)	3.07 ± 0.36	
	≥ 501	26 (20.0)	3.00 ± 0.37	
Living status	Alone	15 (11.5)	3.01 ± 0.49	0.21 (.806)
	With spouse	73 (56.2)	3.02 ± 0.29	
	With family	42 (32.3)	2.97 ± 0.38	
Smoking	Never smoked^a^	58 (44.6)	3.11 ± 0.30	11.93 (< .001)
	Quit smoking^b^	47 (36.2)	3.01 ± 0.37	a,b > c^†^
	Current smoker^c^	25 (19.2)	2.73 ± 0.25	
Alcohol drinking	No	84 (64.6)	3.08 ± 0.37	3.88 (< .001)
	Yes	46 (35.4)	2.86 ± 0.25	
Exercise	No^a^	7 (5.4)	2.75 ± 0.43	9.28 (< .001)
	Irregular^b^	70 (53.8)	2.92 ± 0.33	a,b < c^†^
	Regular^c^	53 (40.8)	3.15 ± 0.31	
BMI (kg/m^2^)	Normal (18.5~22.9)	27 (20.8)	3.06 ± 0.33	1.76 (.175)
	Overweight (23~24.9)	45 (34.6)	2.93 ± 0.36	
	Obesity (≥ 25)	58 (44.6)	3.03 ± 0.34	
Period after PCI (yr)	1~5^a^	78 (60.0)	2.94 ± 0.32	3.51 (.033)
	6~10^b^	40 (30.8)	3.11 ± 0.38	a < b^†^
	≥ 11	12 (9.2)	3.08 ± 0.34	
Restenosis after of PCI	No	112 (86.2)	3.00 ± 0.33	−0.39 (.698)
	Yes	18 (13.8)	3.03 ± 0.43	
Subjective health status	Bad^a^	18 (13.9)	2.85 ± 0.33	7.51 (< .001)
	Moderate^b^	71 (54.6)	2.95 ± 0.34	a,b < c^†^
	Good^c^	41 (31.5)	3.16 ± 0.31	
Diagnosis	MI	44 (33.8)	3.10 ± 0.41	2.08 (.041)
	Angina pectoris	86 (66.2)	2.95 ± 0.30	
Duration of disease (yr)	≤ 1	24 (18.4)	2.98 ± 0.29	2.44 (.067)
	2~5	53 (40.8)	2.92 ± 0.34	
	6~10	39 (30.0)	3.11 ± 0.38	
	≥ 11	14 (10.8)	3.06 ± 0.32	
Comorbidity	Hypertension	58 (44.6)	3.00 ± 0.35	0.56 (.642)
	Diabetes mellitus	30 (23.1)	3.03 ± 0.39	
	Hyperlipidemia	16 (12.3)	2.90 ± 0.20	
	Other (e.g., cerebrovascular disease)	26 (20.0)	3.03 ± 0.36	

M = Mean; SD = Standard deviation; BMI = Body mass index; PCI = Percutaneous coronary intervention; MI = Myocardial infarction.

† Scheffé’s test.

**Table 2.** Degrees of Self-efficacy in Health Management, Resilience, and Treatment Compliance (N = 130)

Variables	M ± SD	Range
Self-efficacy in Health Management	3.75 ± 0.49	1~5
Resilience	3.66 ± 0.45	1~5
Treatment compliance	3.00 ± 0.35	1~4

M = Mean; SD = Standard deviation.

**Table 3.** Correlations among Main Variables (N = 130)

Variables	Self-efficacy in health management	Resilience	Treatment compliance
	r (*p*)	r (*p*)	r (*p*)
Resilience	.63 (< .001)	1	-
Treatment compliance	.46 (< .001)	.64 (< .001)	1

**Table 4.** Factors Influencing Treatment Compliance in Patients with Coronary Artery Disease (N = 130)

Variables	B	SE	β	t (*p*)	Partial R^2^	R^2^	Adjusted R^2^	F (*p*)
Resilience	0.45	0.05	.59	9.04 (< .001)	.41	.41	.45	4.30 (.040)
Alcohol drinking^†^	−0.13	0.04	−.18	−2.84 (.005)	.03	.45		
Diagnosis^‡^	0.10	0.04	.13	2.07 (.040)	.01	.46		

SE = Standard error.

† Alcohol drinking (Yes = 1, No = 0); ^‡^Diagnosis (Myocardial infarction = 1, Angina pectoris = 0).

## Data Availability

The data that support the findings of this study are available from the corresponding author upon reasonable request.
